# Selective Remodeling: Refining Neural Connectivity at the Neuromuscular Junction

**DOI:** 10.1371/journal.pbio.1000185

**Published:** 2009-08-25

**Authors:** Won-Suk Chung, Ben A. Barres

**Affiliations:** Department of Neurobiology, Stanford University School of Medicine, Stanford, California, United States of America

## Abstract

A primer on new research by Fuentes-Medel and colleagues explains the important role of non-neural cells in clearing neural debris, which is continuously produced during the normal remodeling processes that establish and maintain neural connectivity.

The nervous system is an intricately wired communication system that receives and responds to intrinsic and environmental information, allowing the organism to adapt to its surroundings. Proper nervous system function depends on the establishment of correct connectivity between neurons and their target cells. The target cells can be either neurons or non-neuronal peripheral cells, such as muscle cells. The axon of a typical neuron emerges from one end of the main cell body and, in humans, can extend up to several feet to form a connection with a target cell at a specialized site called the synapse. At the synapse, the presynaptic terminal of the axon communicates with target cells through dendrites of neurons or neuromuscular junctions (NMJs) of muscle cells. Therefore, the initial development of neural connectivity involves a series of steps including axonal growth, axonal pathfinding, and synapse formation with the right target cells [1].

In addition to these initial steps, however, extensive remodeling of preformed axons and connections are required to achieve precise neural connectivity. These remodeling processes include the elimination of excess axons, dendrites, synapses, and their debris [2]. Mounting evidence shows that elimination processes are critical in shaping neural circuits during development as well as in regulating synaptic plasticity (the ability of the synapse to change its connection strength) in response to experience and memory [3]. Although recent advances in technology, such as high-resolution imaging of live nervous systems, have helped us to observe the formation and refinement of neural connections, we are just beginning to understand the cellular and molecular mechanisms underlying these phenomena.

## Selective Elimination of Neural Connections during Initial Circuit Shaping and Synaptic Plasticity Regulation

**Figure 1:**
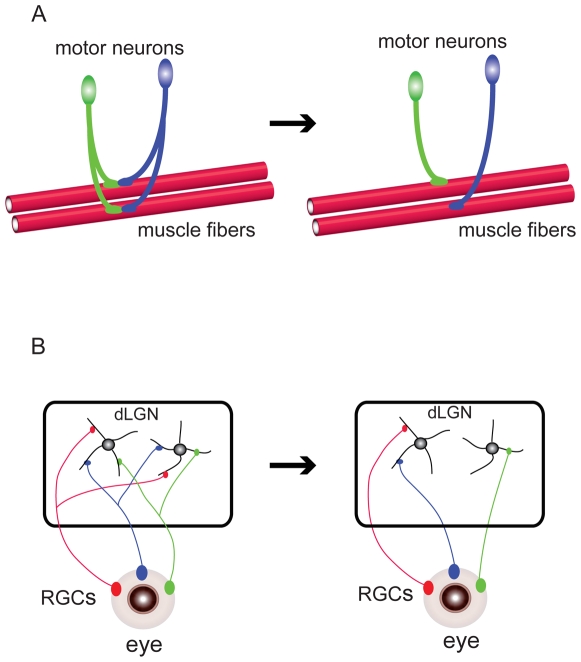
Elimination processes during the shaping of neural circuits. (A) At the mammalian NMJ, axons from motor neurons form connections with muscle fibers. Initially, each NMJ has multiple inputs from two or more motor neurons. However, through activity-dependent intercellular competition, the “loser” axon retracts and is eventually eliminated, leaving a one-to-one match between each motor input and NMJ. (B) In the mammalian retinogeniculate system, eye-specific connections are formed through axonal projections from RGCs to their major target, the dLGN. At an initial stage, a dLGN neuron is multiply innervated by axons originating from many RGCs. Through a competition process driven by neural activity, inappropriate RGC axons are eliminated by selective local degeneration. As a result, each dLGN neuron receives stable inputs from only one or two RGCs.

During the initial phases of neural connectivity, neurons develop exuberant axonal and dendritic processes. These excess processes subsequently undergo selective elimination to shape mature neural circuits. This endeavor may include the local elimination of axons and dendrites through competition between cells for common targets [2],[4]. One well-studied example of this type of neural circuit shaping involves synapse elimination and axonal retraction during neural innervation at the mammalian NMJ (Figure 1A) [5]. Initially, several motor neurons send axons to the same muscle cell, so that one NMJ is innervated by axons from more than one motor neuron. However, within the first several postnatal weeks, all but one of the motor neuron inputs to each NMJ are eliminated, leaving a one-to-one match between each motor input and NMJ. Recent time-lapse imaging has suggested that this elimination of excess axons occurs by retraction of the “loser” axons through a process called axosome shedding, rather than selective degeneration [6]. Likewise, in the visual system of mice (and other mammals as well) (Figure 1B), connections between retinal ganglion cells (RGCs) and their target, the dorsal lateral geniculate nucleus (dLGN), are pruned in a manner that results in each RGC making non-overlapping connections in a target domain [7],[8]. Initially, dLGN neurons are multiply innervated by up to ten RGC axons, which show overlapping axonal branches in the dLGN. However, by the third postnatal week, RGC axons from each eye have been segregated from one another by selective local degeneration. As a result, each dLGN neuron receives stable inputs from only one or two RGC axons.

As these two examples of remodeling processes illustrate, entire exuberant axon branches can be eliminated by either local retraction or degeneration. Neural circuits can also be remodeled on a much finer scale during synaptic plasticity regulation.

**Figure 2:**
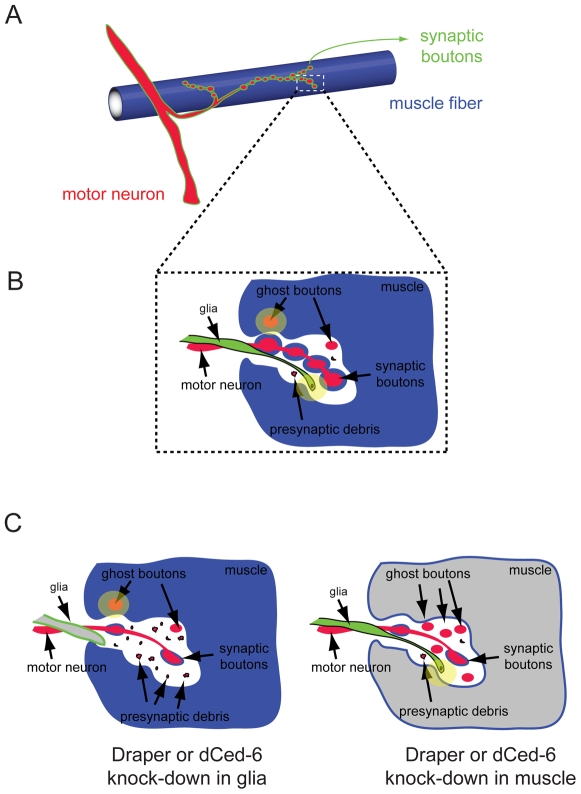
Elimination processes at the *Drosophila* NMJ in synaptic plasticity regulation. (A) At the *Drosophila* NMJ, a single arbor from the motor neuron (red) innervates a muscle fiber (blue) and forms synaptic boutons (green), at the site at which the presynaptic terminal of the axon communicates with the postsynaptic site on the muscle cell. (B) A new study from Fuentes-Medel et al. shows that, in response to changes in growth and/or activity, the addition of new synaptic connections with the muscle cell involves significant production of presynaptic debris and ghost boutons. The presynaptic debris and ghost boutons are engulfed and eliminated by glial and muscle cells, respectively (light yellow circles). (C) Knocking down Draper or dCed-6 function in glia results in the accumulation of presynaptic debris, whereas clearance of ghost boutons by muscle cells is intact. In contrast, blocking muscle-mediated phagocytosis causes the accumulation of ghost boutons without affecting presynaptic debris clearance by glia. Disruption of either one of these neural debris clearance processes is sufficient to interfere with proper formation of synaptic boutons and leads to severely compromised synaptic growth.

During synaptic plasticity regulation, the addition/growth and elimination of synapses within a single neural branch modulate connectivity between the presynaptic terminal of the axon and the postsynaptic site of the target cell. In these processes, changes in electrical activity result in changes in synaptic efficacy, often accompanied by structural changes in the synapses themselves. For example, at the *Drosophila* larval NMJ, new synapses and synaptic boutons (a button-like swollen end of an axon at a synapse) are constantly formed and stabilized as the target muscle cells grow in size [9]. This coordinated increase between synapses and muscle size serves to maintain synaptic efficacy during the expansion of muscle fibers. Interestingly, in this issue of *PLoS Biology*, Yuly Fuentes-Medel et al. [10] show that the addition of new synapses at the *Drosophila* NMJ involves significant production of presynaptic membrane debris and detachment of undifferentiated synaptic boutons (“ghost boutons”) (Figures 2A and 2B). Ghost boutons are devoid of pre- and postsynaptic compartments, although they contain some elements of a synapse, such as synaptic vesicles, suggesting an undifferentiated bouton state [11]. In previous studies, these ghost boutons have been found in the normal NMJ at very low frequency and have been shown to give rise to mature boutons [12]. Also, significant increases in their formation have been observed after motor neuron stimulation [12]. These authors confirmed that ghost boutons were able to mature and differentiate. Then, building on this finding through the use of careful time-lapse imaging of intact larvae with light-controlled activity stimulation, Fuentes-Medel et al. noticed that significant portions of the ghost boutons failed to mature and eventually disappeared over time. Along with the ghost boutons, the amount of presynaptic membrane debris significantly increased after stimulating motor neurons, independent of new ghost bouton formation. These results convincingly show that the remodeling of the *Drosophila* NMJ involves continuous shedding and elimination of certain presynaptic membrane compartments.

## The Cellular and Molecular Players of Neural Debris Clearance

How is neural debris cleared away and what would be the significance of this mechanism? Studies in various species, including mammals and flies, have discovered that a population of non-neuronal cells known as glial cells play central roles in clearing neural debris through an engulfment process called phagocytosis [13],[14]. This phagocytic process involves the proper recognition by glial cells, ingestion, and degradation of the neural debris. For example, in the mammalian nervous system, microglia (a resident population of professional phagocytes) in the brain [15] and Schwann cells (glial cells that ensheathe peripheral axons) at the NMJ [5],[6] have been shown to clear neural debris during development as well as following injury. In response to brain injury, microglia cells are activated and shield injury sites in the course of clearing dying (“apoptotic”) neurons [15]. Recently, it has been suggested that microglia also participate in eliminating excess axons and synapses in the developing dLGN through both the classical complement cascade (a biochemical cascade that helps clear pathogens from an organism as a part of an immune system) and other, as-yet-unidentified mechanisms [16].

As in the mammalian nervous system, glial cells in *Drosophila* again turn out to be the main cell type responsible for eliminating excess axons during development [14],[17] and clearing severed, degenerating axons during injury [13]. Importantly, genetic studies involving worms, flies, and rodents have identified a number of genes required for glial cells to clear cellular debris [18]–[20]. Those genes fall into at least three, partially redundant pathways that activate phagocytosis [21]. The first pathway includes the proteins Ced-2 (an ortholog of mammalian CrKII), Ced-5 (DOCK 180), Ced-10 (Rac1), and Ced-12 (ELMO), and controls rearrangement of the actin cytoskeleton, which is required to surround the cellular debris. A recent study has also identified Bai1 as a receptor acting upstream of these components [22]. The second pathway includes the c-Mer tyrosine kinase receptor (MerTK), which works with the Integrin pathway to regulate CrKII/DOCK 180/Rac1 modules [20],[23]. The last pathway consists of Ced-1 (an ortholog of fly Draper, a phagocytic receptor), Ced-6 (an ortholog of mammalian GULP, an adaptor protein), and Ced-7 (an ABC transporter), and participates in cellular debris recognition and engulfment [24]. Multiple studies disrupting Draper function in the fly have revealed that Draper is involved in most or all elimination processes including the engulfment of apoptotic neurons, the elimination of excess axons during fly development [25], and the elimination of severed axons in the olfactory system [13].

Now, with these new findings from Fuentes-Medel et al., glial cells at the *Drosophila* NMJ have also been shown to clear synaptic debris, thereby helping to control synaptic connectivity within a single arbor. Glial cells were found to cover the NMJ and extend highly dynamic membrane projections to engulf presynaptic debris (Figure 2B). Glial cells' phagocytic activity was dependent on Draper and dCed-6 (a fly ortholog of worm Ced-6), because specific knock-down of either of the proteins in glial cells resulted in the significant accumulation of presynaptic debris (Figure 2C). Surprisingly, Fuentes-Medel et al. found that muscle cells also express Draper. This novel finding led them to test whether muscle cells cooperate in clearing the presynaptic material. Indeed, when Draper and dCed-6 were knocked down in muscle cells, flies showed defects in clearing neural debris. Remarkably, however, each cell type seems to have a distinct function during the engulfment process; glial cells primarily engulf presynaptic debris, whereas muscle cells primarily engulf ghost boutons (Figure 2C). This observation strongly suggests that muscle cells are not simply postsynaptic target cells, but tissue resident phagocytes that participate in sculpting the *Drosophila* NMJ.

Importantly, the new findings of Fuentes-Medel et al. reveal the functional significance of these neural clearing mechanisms. Disruption of phagocytic activity either in glial or muscle cells caused the accumulation of presynaptic debris and ghost boutons, respectively, resulting in a severely reduced number of synaptic boutons and boutons displaying abnormal growth (Figure 2C). This finding implies that normal synaptic growth at the NMJ continuously produces presynaptic debris and ghost boutons in response to changes in growth and activity. Failure of glial and muscle cells to clear the accumulating debris interferes with proper formation of synaptic boutons and subsequent synaptic connectivity.

These new findings from Fuentes-Medel et al. raise several exciting questions. Why do glial and muscle cells have different effects in clearing neural debris? Does this simply reflect the fact that glial cells work at the NMJ with very thin membrane projections, so that they can only catch smaller debris? Or are there differences in molecular mechanisms, such that the presynaptic debris and ghost boutons are recognized in molecularly distinct ways? It is clear that Draper is required in clearing presynaptic debris and ghost boutons, implying that similar “eat me” signals may be present in both cases. Identifying these “eat me” signals that tag specific neural materials for phagocytic uptake is a critical goal for future studies. Given the fact that the *Drosophila* NMJ continuously produces presynaptic remnants that require clearing to regulate synaptic connectivity, it is tempting to speculate that this process could be a more general phenomenon in many other synaptic connections. It would therefore be interesting to investigate whether synaptic connections in the mammalian NMJ or brain exhibit similar pre- or postsynaptic membrane shedding and subsequent clearance upon changes in synaptic plasticity.

The current repertoire of tissue resident phagocytes is likely to expand based on several studies [26] including the one from Fuentes-Medel et al. Since eliminating various cellular components (from small membrane debris to the entire cell body) is crucial not only during injury states but also during normal physiological states, having a variety of tissue resident phagocytes ensures robust clearing of cellular debris in response to rapid changes. For example, in mammals, growing evidence suggests that astrocytes, another glial subtype in the brain, may also play a role in clearing neural debris [27]–[29]. It is possible that these new players do their job in coordination with professional phagocytes, such as macrophages and microglia. How they coordinate the elimination process of the neural debris and whether there is any specificity in recognizing the target debris are now questions that beg further investigation.

## Abbreviations

dLGN: dorsal lateral geniculate nucleus
NMJ: neuromuscular junction
RGC: retinal ganglion cell

## References

[1] O'Donnell M, Chance R, Bashaw G (2009). Axon growth and guidance: Receptor regulation and signal transduction.. Annu Rev Neurosci.

[2] Luo L, O'Leary D (2005). Axon retraction and degeneration in development and disease.. Annu Rev Neurosci.

[3] Alvarez V, Sabatini B (2007). Anatomical and physiological plasticity of dendritic spines.. Annu Rev Neurosci.

[4] Low L, Cheng H (2005). A little nip and tuck: Axon refinement during development and axonal injury.. Curr Opin Neurobiol.

[5] Walsh M, Lichtman J (2003). In vivo time-lapse imaging of synaptic takeover associated with naturally occurring synapse elimination.. Neuron.

[6] Bishop D, Misgeld T, Walsh M, Gan W, Lichtman J (2004). Axon branch removal at developing synapses by axosome shedding.. Neuron.

[7] Hooks B, Chen C (2006). Distinct roles for spontaneous and visual activity in remodeling of the retinogeniculate synapse.. Neuron.

[8] Huberman A (2007). Mechanisms of eye-specific visual circuit development.. Curr Opin Neurobiol.

[9] Schuster C, Davis G, Fetter R, Goodman C (1996). Genetic dissection of structural and functional components of synaptic plasticity. II. Fasciclin II controls presynaptic structural plasticity.. Neuron.

[10] Fuentes-Medel Y, Logan M. A, Ashley J, Ataman B, Budnik V (2009). Glia and muscle sculpt neuromuscular arbors by engulfing destabilized synaptic boutons and shed presynaptic debris.. PLoS Biol.

[11] Ataman B, Ashley J, Gorczyca D, Gorczyca M, Mathew D (2006). Nuclear trafficking of *Drosophila* Frizzled-2 during synapse development requires the PDZ protein dGRIP.. Proc Natl Acad Sci U S A.

[12] Ataman B, Ashley J, Gorczyca M, Ramachandran P, Fouquet W (2008). Rapid activity-dependent modifications in synaptic structure and function require bidirectional Wnt signaling.. Neuron.

[13] MacDonald J, Beach M, Porpiglia E, Sheehan A, Watts R (2006). The *Drosophila* cell corpse engulfment receptor Draper mediates glial clearance of severed axons.. Neuron.

[14] Watts R, Schuldiner O, Perrino J, Larsen C, Luo L (2004). Glia engulf degenerating axons during developmental axon pruning.. Curr Biol.

[15] Nimmerjahn A, Kirchhoff F, Helmchen F (2005). Resting microglial cells are highly dynamic surveillants of brain parenchyma in vivo.. Science.

[16] Stevens B, Allen N, Vazquez L, Howell G, Christopherson K (2007). The classical complement cascade mediates CNS synapse elimination.. Cell.

[17] Kurant E, Axelrod S, Leaman D, Gaul U (2008). Six-microns-under acts upstream of Draper in the glial phagocytosis of apoptotic neurons.. Cell.

[18] Mangahas P, Zhou Z (2005). Clearance of apoptotic cells in *Caenorhabditis elegans*.. Semin Cell Dev Biol.

[19] Zhou Z, Hartwieg E, Horvitz H (2001). CED-1 is a transmembrane receptor that mediates cell corpse engulfment in *C. elegans*.. Cell.

[20] D'Cruz P, Yasumura D, Weir J, Matthes M, Abderrahim H (2000). Mutation of the receptor tyrosine kinase gene Mertk in the retinal dystrophic RCS rat.. Hum Mol Genet.

[21] Mallat M, Marín-Teva J, Chéret C (2005). Phagocytosis in the developing CNS: More than clearing the corpses.. Curr Opin Neurobiol.

[22] Park D, Tosello-Trampont A, Elliott M, Lu M, Haney L (2007). BAI1 is an engulfment receptor for apoptotic cells upstream of the ELMO/Dock180/Rac module.. Nature.

[23] Wu Y, Singh S, Georgescu M, Birge R (2005). A role for Mer tyrosine kinase in alphavbeta5 integrin-mediated phagocytosis of apoptotic cells.. J Cell Sci.

[24] Yu X, Lu N, Zhou Z (2008). Phagocytic receptor CED-1 initiates a signaling pathway for degrading engulfed apoptotic cells.. PLoS Biol.

[25] Hoopfer E, McLaughlin T, Watts R, Schuldiner O, O'Leary D (2006). Wlds protection distinguishes axon degeneration following injury from naturally occurring developmental pruning.. Neuron.

[26] Bronson R (1998). Is the oocyte a non-professional phagocyte?. Hum Reprod Update.

[27] Cahoy J, Emery B, Kaushal A, Foo L, Zamanian J (2008). A transcriptome database for astrocytes, neurons, and oligodendrocytes: A new resource for understanding brain development and function.. J Neurosci.

[28] Bechmann I, Nitsch R (1997). Astrocytes and microglial cells incorporate degenerating fibers following entorhinal lesion: A light, confocal, and electron microscopical study using a phagocytosis-dependent labeling technique.. Glia.

[29] Tansey F, Cammer W (1998). Differential uptake of dextran beads by astrocytes, macrophages and oligodendrocytes in mixed glial-cell cultures from brains of neonatal rats.. Neurosci Lett.

